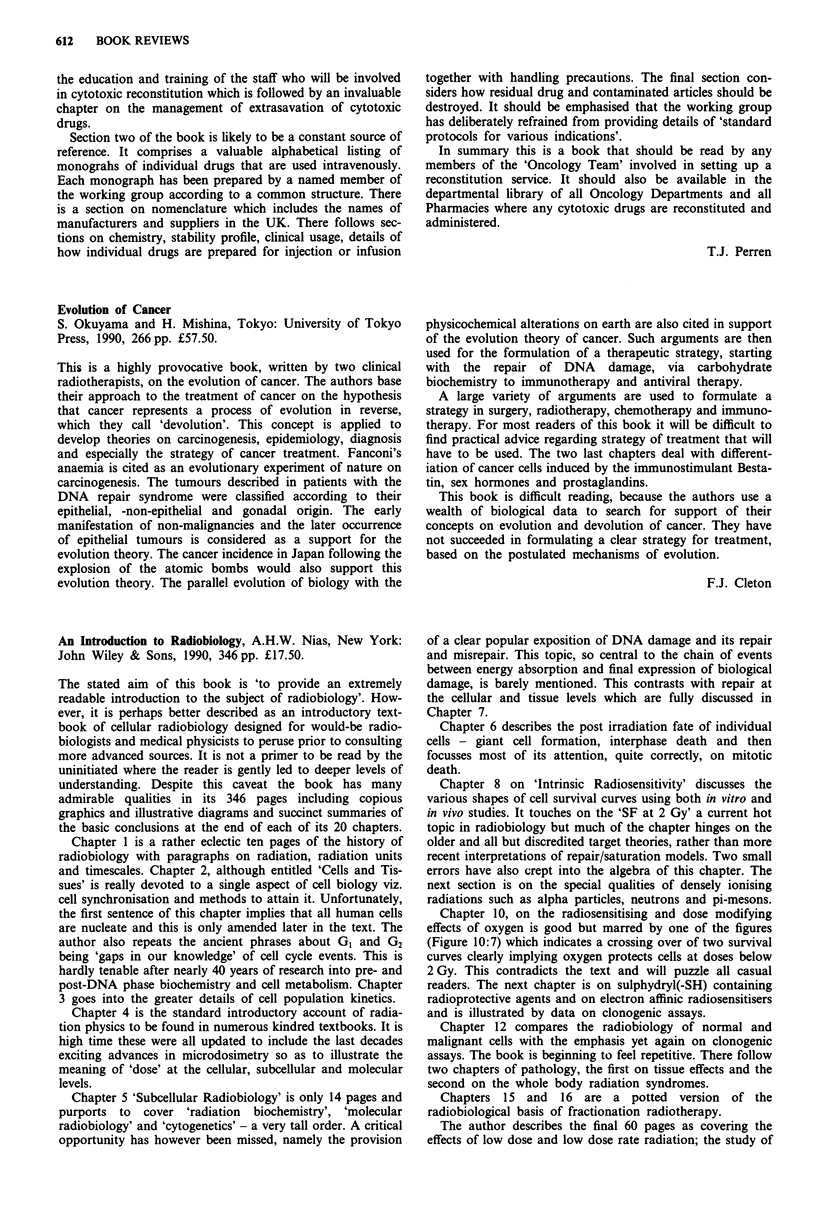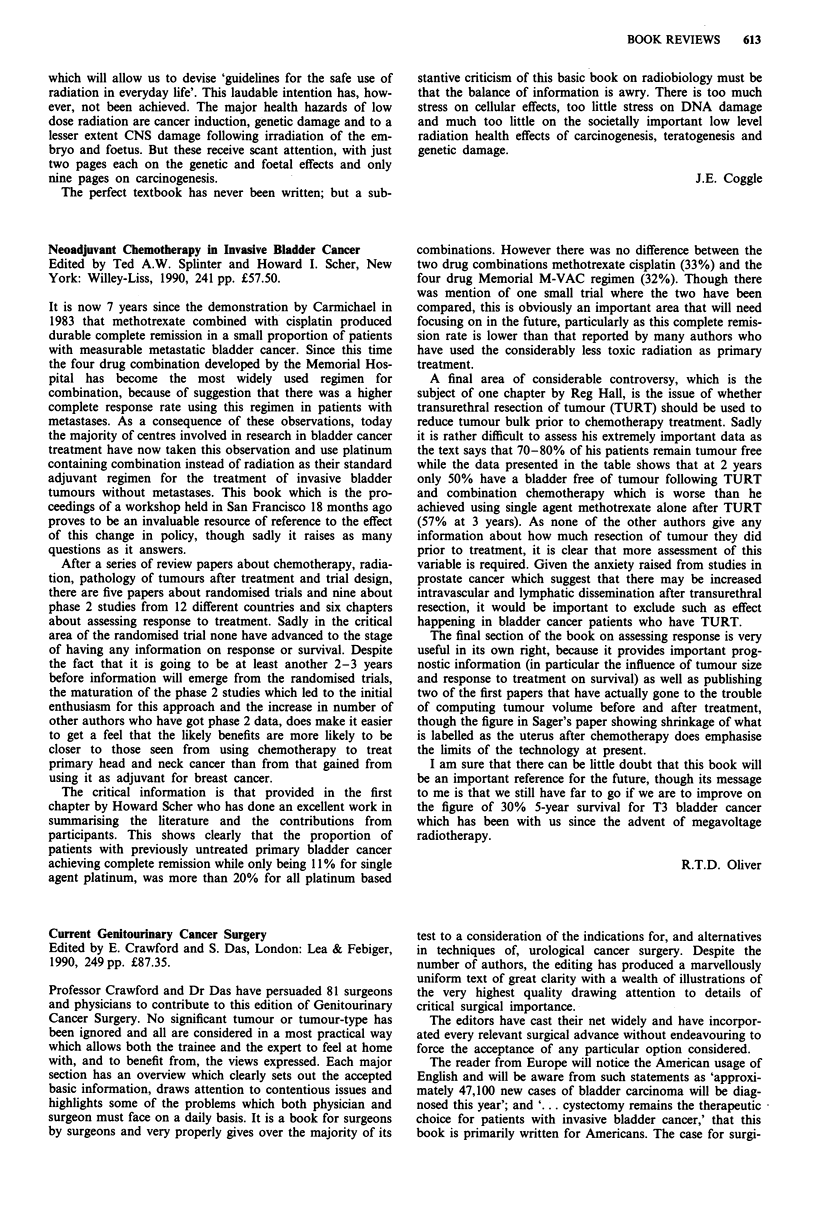# An Introduction to Radiobiology

**Published:** 1991-09

**Authors:** J.E. Coggle


**An Introduction to Radiobiology**, A.H.W. Nias, New York: John Wiley & Sons, 1990, 346 pp. £17.50.

The stated aim of this book is 'to provide an extremely readable introduction to the subject of radiobiology'. However, it is perhaps better described as an introductory textbook of cellular radiobiology designed for would-be radiobiologists and medical physicists to peruse prior to consulting more advanced sources. It is not a primer to be read by the uninitiated where the reader is gently led to deeper levels of understanding. Despite this caveat the book has many admirable qualities in its 346 pages including copious graphics and illustrative diagrams and succinct summaries of the basic conclusions at the end of each of its 20 chapters.

Chapter 1 is a rather eclectic ten pages of the history of radiobiology with paragraphs on radiation, radiation units and timescales. Chapter 2, although entitled 'Cells and Tissues' is really devoted to a single aspect of cell biology viz. cell synchronisation and methods to attain it. Unfortunately, the first sentence of this chapter implies that all human cells are nucleate and this is only amended later in the text. The author also repeats the ancient phrases about $G_1$ and $G_2$ being 'gaps in our knowledge' of cell cycle events. This is hardly tenable after nearly 40 years of research into pre- and post-DNA phase biochemistry and cell metabolism. Chapter 3 goes into the greater details of cell population kinetics.

Chapter 4 is the standard introductory account of radiation physics to be found in numerous kindred textbooks. It is high time these were all updated to include the last decades exciting advances in microdosimetry so as to illustrate the meaning of 'dose' at the cellular, subcellular and molecular levels.

Chapter 5 'Subcellular Radiobiology' is only 14 pages and purports to cover 'radiation biochemistry', 'molecular radiobiology' and 'cytogenetics' – a very tall order. A critical opportunity has however been missed, namely the provision of a clear popular exposition of DNA damage and its repair and misrepair. This topic, so central to the chain of events between energy absorption and final expression of biological damage, is barely mentioned. This contrasts with repair at the cellular and tissue levels which are fully discussed in Chapter 7.

Chapter 6 describes the post irradiation fate of individual cells – giant cell formation, interphase death and then focusses most of its attention, quite correctly, on mitotic death.

Chapter 8 on 'Intrinsic Radiosensitivity' discusses the various shapes of cell survival curves using both *in vitro* and *in vivo* studies. It touches on the 'SF at 2 Gy' a current hot topic in radiobiology but much of the chapter hinges on the older and all but discredited target theories, rather than more recent interpretations of repair/saturation models. Two small errors have also crept into the algebra of this chapter. The next section is on the special qualities of densely ionising radiations such as alpha particles, neutrons and pi-mesons.

Chapter 10, on the radiosensitising and dose modifying effects of oxygen is good but marred by one of the figures (Figure 10:7) which indicates a crossing over of two survival curves clearly implying oxygen protects cells at doses below 2 Gy. This contradicts the text and will puzzle all casual readers. The next chapter is on sulphydryl(-SH) containing radioprotective agents and on electron affinic radiosensitisers and is illustrated by data on clonogenic assays.

Chapter 12 compares the radiobiology of normal and malignant cells with the emphasis yet again on clonogenic assays. The book is beginning to feel repetitive. There follow two chapters of pathology, the first on tissue effects and the second on the whole body radiation syndromes.

Chapters 15 and 16 are a potted version of the radiobiological basis of fractionation radiotherapy.

The author describes the final 60 pages as covering the effects of low dose and low dose rate radiation; the study of which will allow us to devise 'guidelines for the safe use of radiation in everyday life'. This laudable intention has, however, not been achieved. The major health hazards of low dose radiation are cancer induction, genetic damage and to a lesser extent CNS damage following irradiation of the embryo and foetus. But these receive scant attention, with just two pages each on the genetic and foetal effects and only nine pages on carcinogenesis.

The perfect textbook has never been written; but a substantive criticism of this basic book on radiobiology must be that the balance of information is awry. There is too much stress on cellular effects, too little stress on DNA damage and much too little on the societally important low level radiation health effects of carcinogenesis, teratogenesis and genetic damage.

J.E. Coggle